# Supplementary material for: Solvent-triggered reversible interconversion of all-nitrogen-donor-protected silver nanoclusters and their responsive optical properties
Source: Nat Commun. 2019 Sep 6;10:4032. doi: 10.1038/s41467-019-11988-y (PMC6731268; doi:10.1038/s41467-019-11988-y)
Supplement: Supplementary file 1 — Supporting Information [file 41467_2019_11988_MOESM1_ESM.pdf]

Supporting Information

Solvent-triggered reversible interconversion of all-nitrogen-donor-protected silver nanoclusters and their responsive optical properties

Yuan et al.

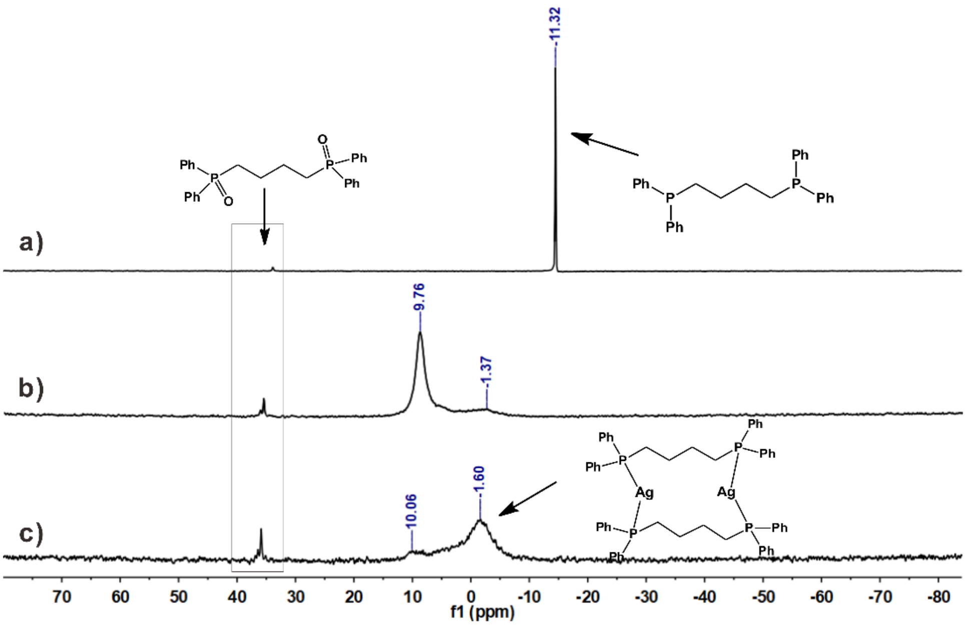

Supplementary Figure 1. Trace of phosphine ligands with ^{31}P NMR in solution. (a) Free dppb in CH_2Cl_2. The result solution before (b) and after adding $NaBH_4$ overnight (c) in the synthesis of **Ag_{22}**. Note, the weak signal of dppb oxide were observed, which were oxidized by air.

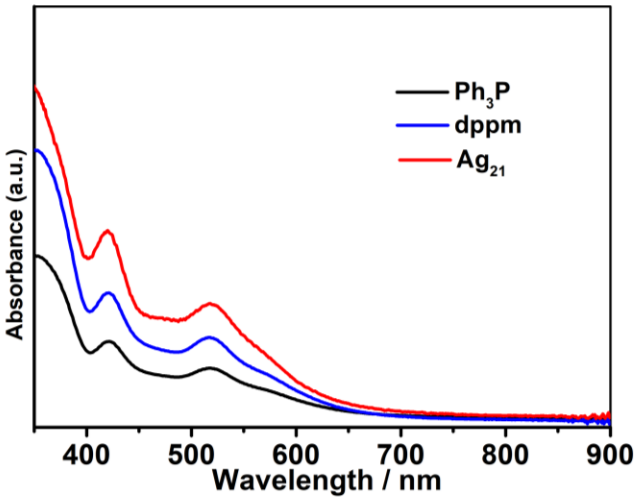

Supplementary Figure 2. Comparison of optical absorption spectra of the **Ag_{21}** crystal sample prepared by dppb and the crude products in the presence of other different phosphine ligands.

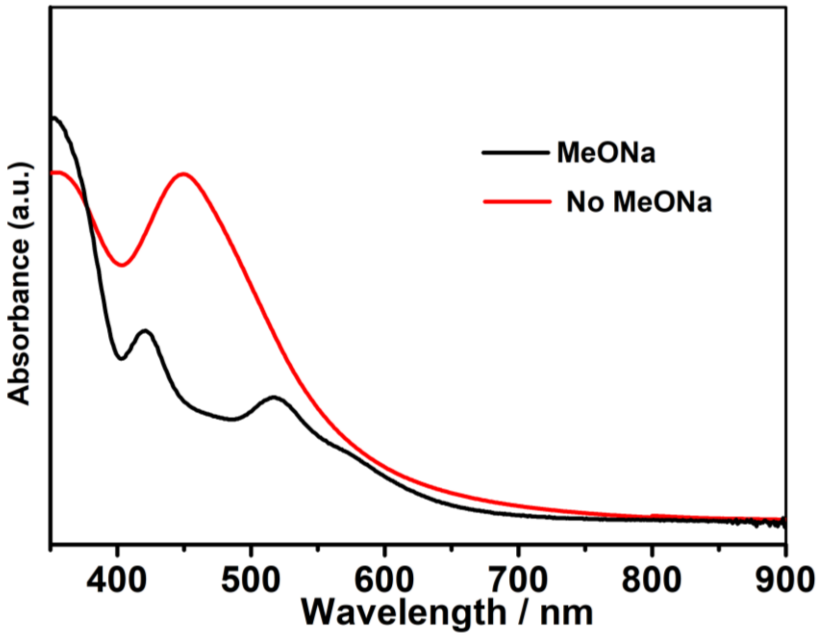

Supplementary Figure 3. Comparison of optical absorption spectra of the crude products with and without MeONa.

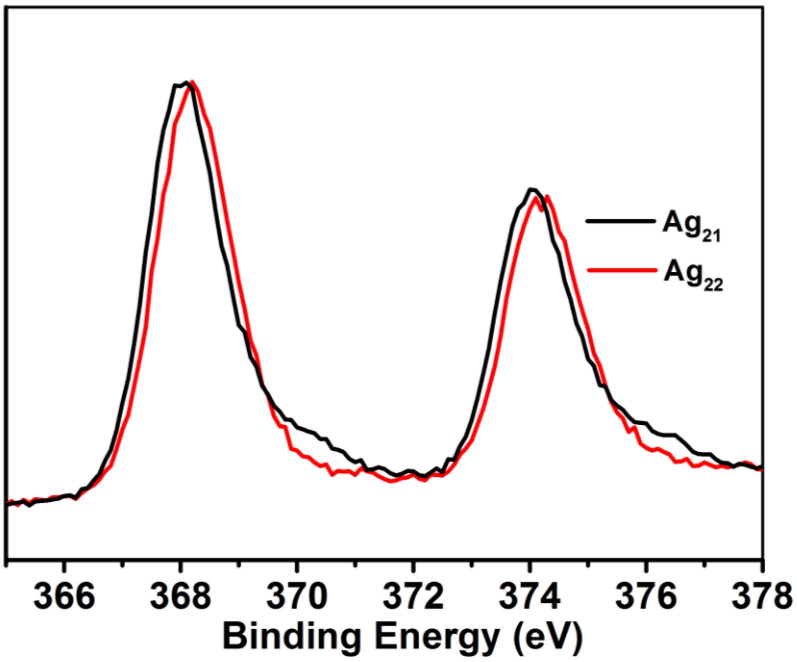

Supplementary Figure 4. XPS spectrum of $\mathbf{Ag_{21}}$ (black profile) and $\mathbf{Ag_{22}}$ (red profile) nanoclusters.

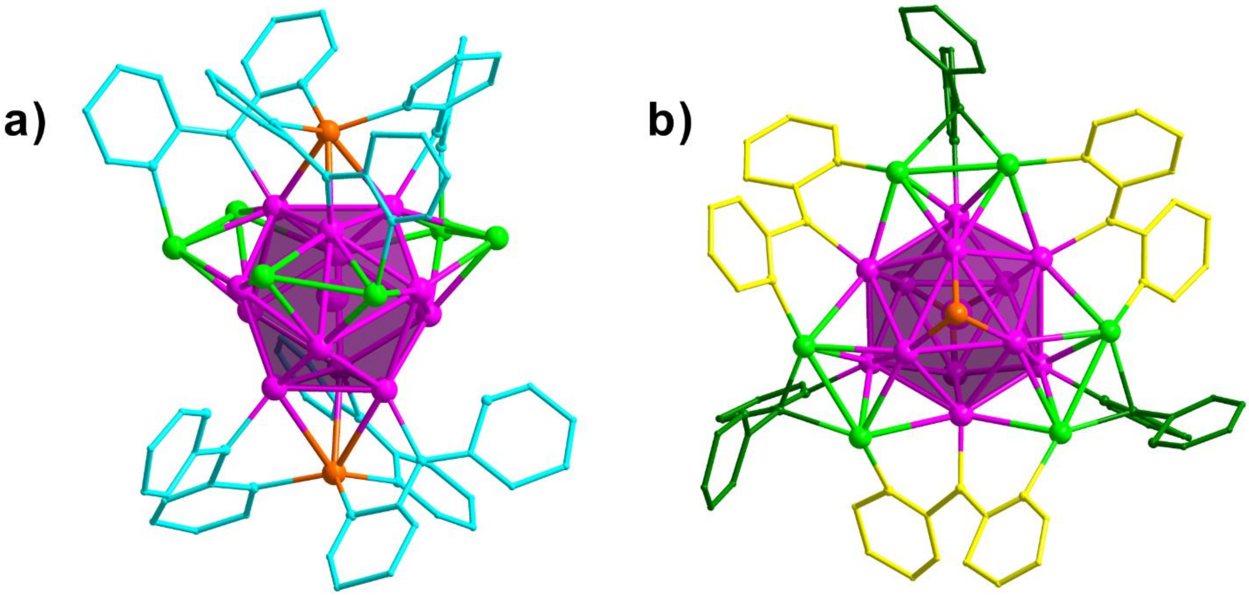

Supplementary Figure 5. The surface structures of $\mathbf{Ag_{21}}$ cluster. (a) Side view and (b) top view of the complex with Ag_{13} core and eight faces capped silver atoms. Color codes: purple = Ag atoms of Ag_{13} core, bright green = cap Ag atoms; surface dpa ligands are colored blue, green and yellow.

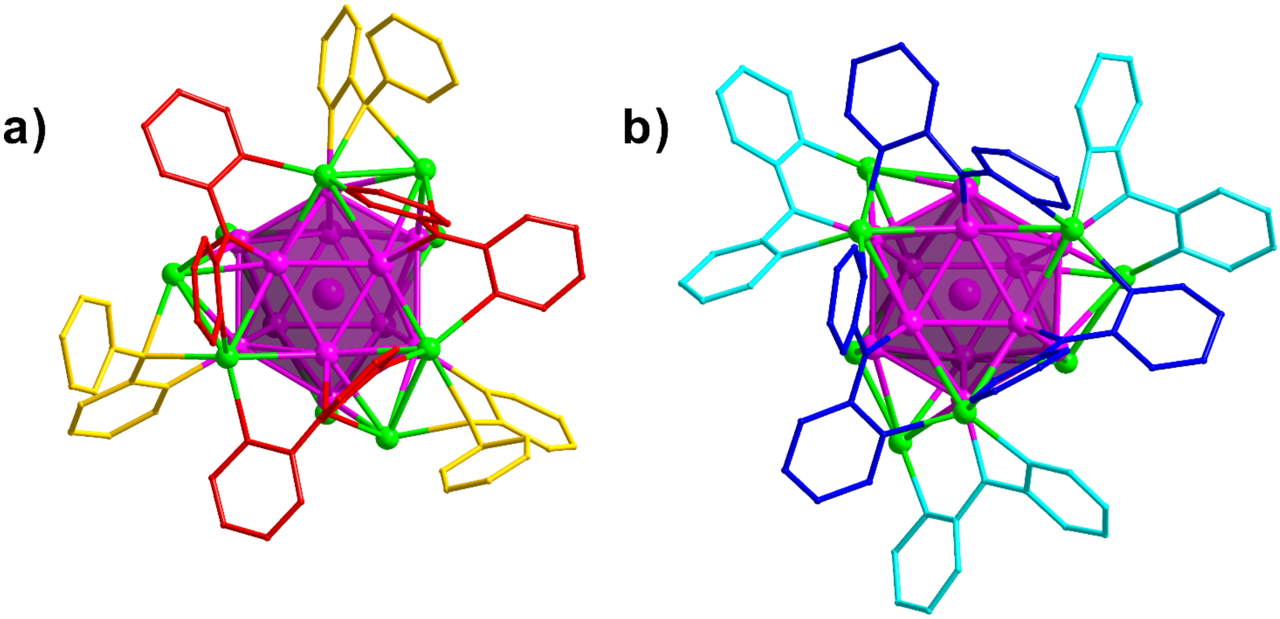

Supplementary Figure 6. The surface structures of $\mathbf{Ag_{22}}$ cluster. (A) Top view and (B) down view of the complex with Ag_{13} core and nine faces capped silver atoms. Color codes: purple = Ag atoms of Ag_{13} core, bright green = cap Ag atoms; surface dpa ligands are colored blue, light blue, red and yellow.

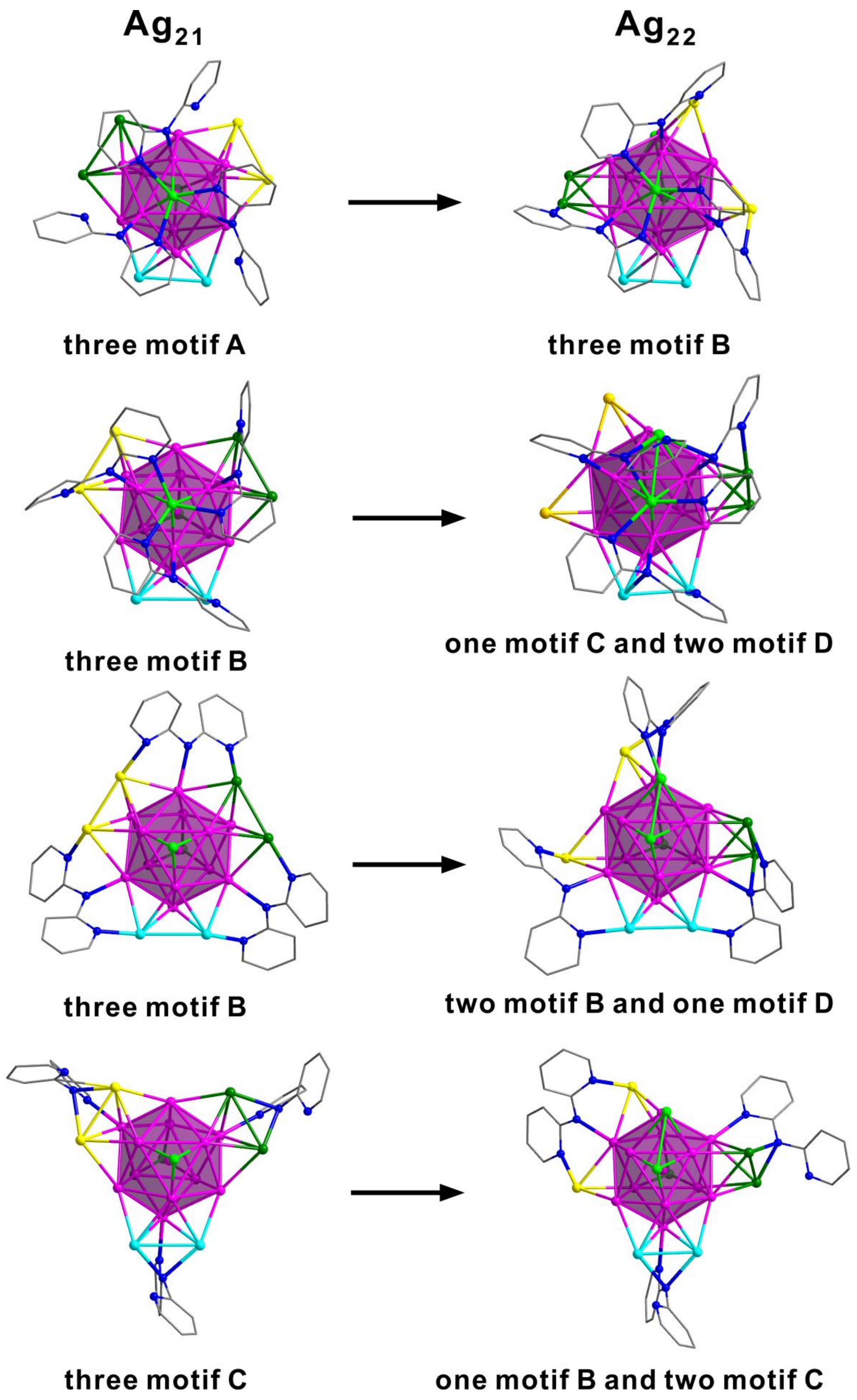

Supplementary Figure 7. Surface structural comparison of $[Ag_{21}(dpa)_{12}]^{+}$ and $[Ag_{22}(dpa)_{12}]^{2+}$ in detail. Color codes: purple sphere, Ag atoms of Ag_{13} core; blue sphere, N; gray sphere, C; cap silver atoms are colored four groups of green, yellow, bright green and light brue.

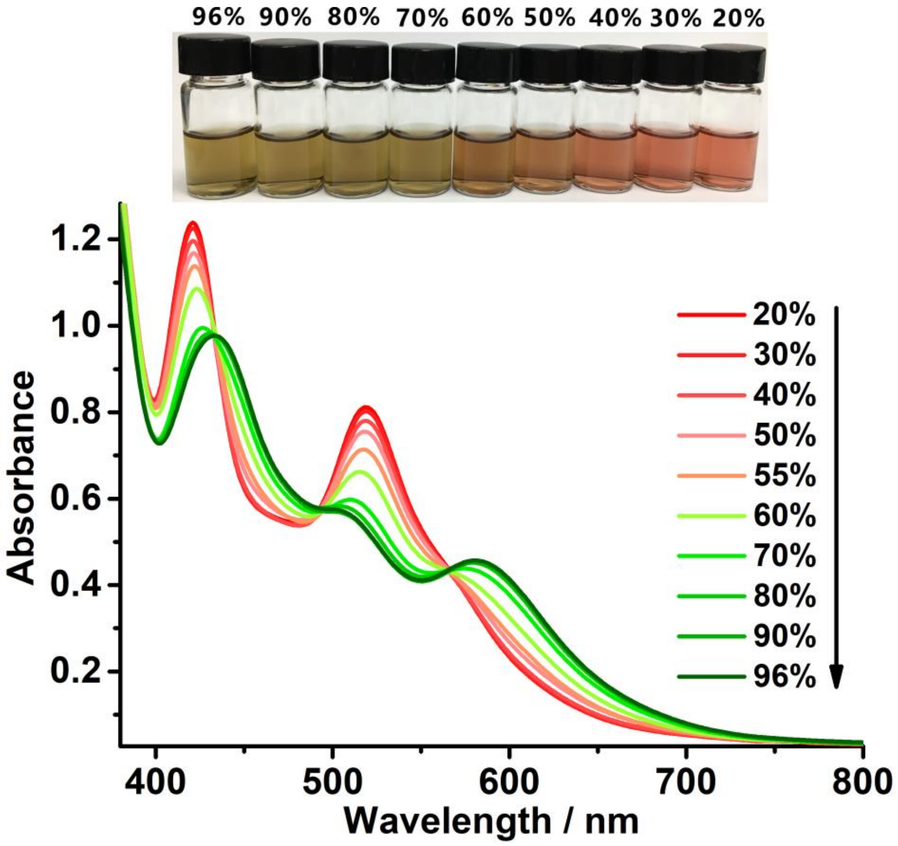

Supplementary Figure 8. The optical absorption spectra of the transformation from $\mathbf{Ag_{21}}$ to $\mathbf{Ag_{22}}$ with different volume ratios of EtOH and n-hexane.

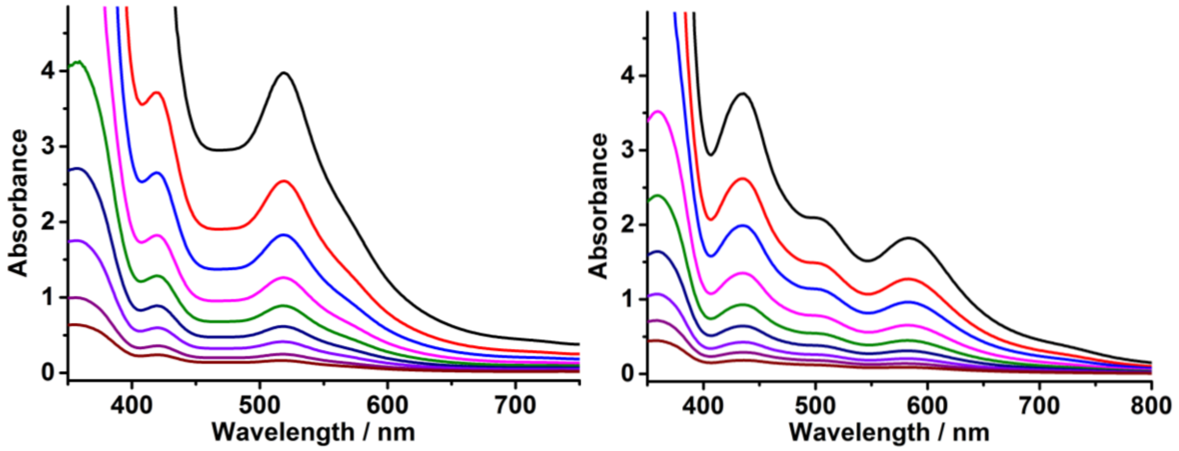

Supplementary Figure 9. UV/vis absorption spectra of **Ag$_{21}$** (left) and **Ag$_{22}$** (right) of various concentrations in EtOH.

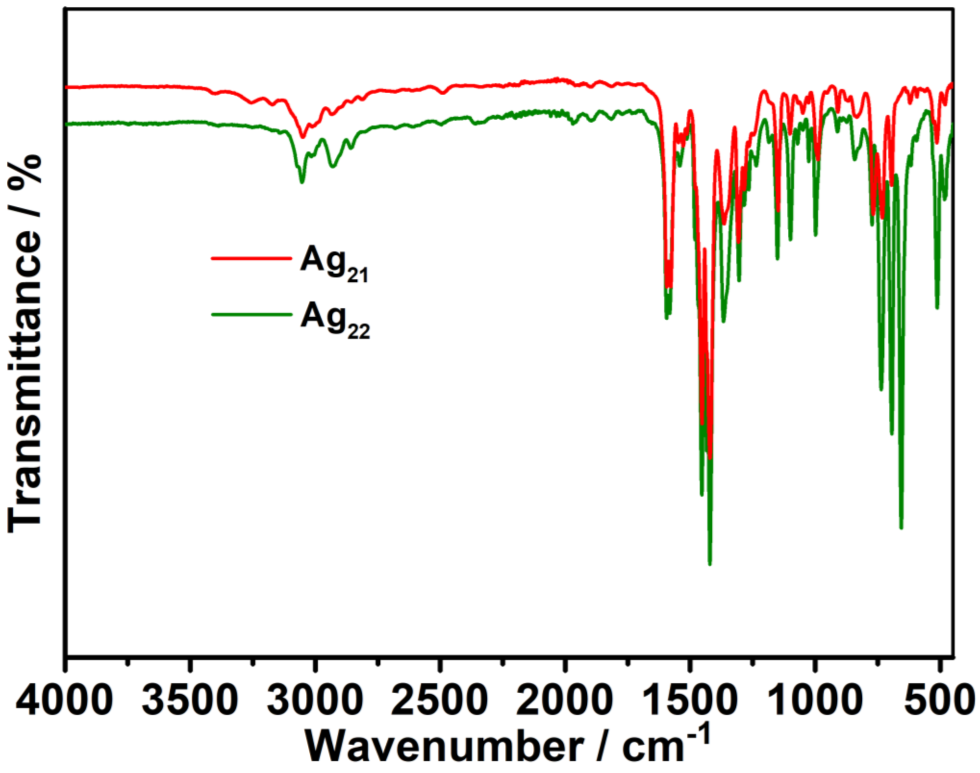

Supplementary Figure 10. FT-IR spectra of **Ag$_{21}$** and **Ag$_{22}$**.

Supplementary Table1. Selected bonds lengths of **Ag_{21}** and **Ag_{22}** clusters.

Bonds	**Ag_{21}** (Å)	**Ag_{22}** (Å)
Internal Ag-Ag in Ag_{13}	2.790 (2.757-2.832)	2.787 (2.743-2.833)
Peripheral Ag-Ag in Ag_{13}	2.933 (2.770-3.168)	2.933 (2.791-3.097)
Capping Ag to Ag_{13} core	2.867 (2.715-2.975)	2.901 (2.767-3.075)
Between capping Ag atoms	3.054 (2.971-3.112)	2.912 (2.909-2.915)
Amido N to Ag atoms	2.256 (2.042-2.510)	2.308 (2.210-2.514)
Pyridyl N to Ag atoms	2.242 (2.108-2.371)	2.255 (2.165-2.334)

Supplementary Table 2. Crystal data and structure refinement for **Ag_{21}**.

Empirical formula	$C_{250}H_{212}N_{72}F_{12}Cl_{20}Ag_{42}Sb_2$
Formula weight	9935.94
Temperature/K	173.4(7)
Crystal system	triclinic
Space group	*P*1
Unit cell dimensions	a = 15.6195(7) Å α = 95.839(3) ° b = 18.2852(7) Å β = 90.418(3) ° c = 26.5537(9) Å γ = 90.042(3) °
Volume/Å^3	7544.3(5)
Z	1
Calculated density (g/cm^3)	2.187
Absorption coefficient /mm^{-1}	24.856
F(000)	4740.0
Crystal size/mm^3	0.15 × 0.04 × 0.03
Radiation	Cu Kα (λ = 1.54178)
2Θ range for data collection/°	6.596 to 121.208
Index ranges	-16 ≤ h ≤ 17, -19 ≤ k ≤ 20, -26 ≤ l ≤ 29
Reflections collected	39559
Independent reflections	25481 [R_{int} = 0.1233, R_{sigma} = 0.1819]
Data/restraints/parameters	25481/26/1757
Goodness-of-fit on F^2	1.029
Final R indexes [I>=2σ (I)]	R_1 = 0.1127, wR_2 = 0.2766
Final R indexes [all data]	R_1 = 0.1253, wR_2 = 0.3011
Largest diff. peak/hole / e Å^{-3}	4.14/-3.59

Supplementary Table 3. Crystal data and structure refinement for **Ag_{22}**.

Empirical formula	$C_{130}H_{112}N_{36}OF_{12}Cl_4Ag_{22}Sb_2$
Formula weight	5180.99
Temperature/K	173.00(10)
Crystal system	monoclinic
Space group	$P2_1$/c
a/Å Unit cell dimensions	a = 28.6773(4) Å α = 90 °<br>b = 16.9550(2) Å β = 96.447(2) °<br>c = 33.7887(5) Å γ = 90 °
Volume/$Å^3$	16325.0(4)
Z	4
Calculated density (g/cm^3)	2.108
Absorption coefficient /mm^{-1}	24.371
F(000)	9856.0
Crystal size/mm^3	0.08 × 0.06 × 0.05
Radiation	CuKα (λ = 1.54184)
2Θ range for data collection/°	7.41 to 121.996
Index ranges	-27 ≤ h ≤ 32, -18 ≤ k ≤ 19, -38 ≤ l ≤ 37
Reflections collected	77432
Independent reflections	24579 [R_{int} = 0.0849, R_{sigma} = 0.0807]
Data/restraints/parameters	24579/969/1819
Goodness-of-fit on F^2	1.038
Final R indexes [I>=2σ (I)]	R_1 = 0.1412, wR_2 = 0.3748
Final R indexes [all data]	R_1 = 0.1532, wR_2 = 0.3844
Largest diff. peak/hole / e $Å^{-3}$	4.96/-3.10
